# Supplementary material for: Evaluation of Polyamine Transport Inhibitors in a Drosophila Epithelial Model Suggests the Existence of Multiple Transport Systems
Source: Med Sci (Basel). 2017 Nov 14;5(4):27. doi: 10.3390/medsci5040027 (PMC5753656; doi:10.3390/medsci5040027)
Supplement: Supplementary file 1 [file medsci-05-00027-s001.pdf]

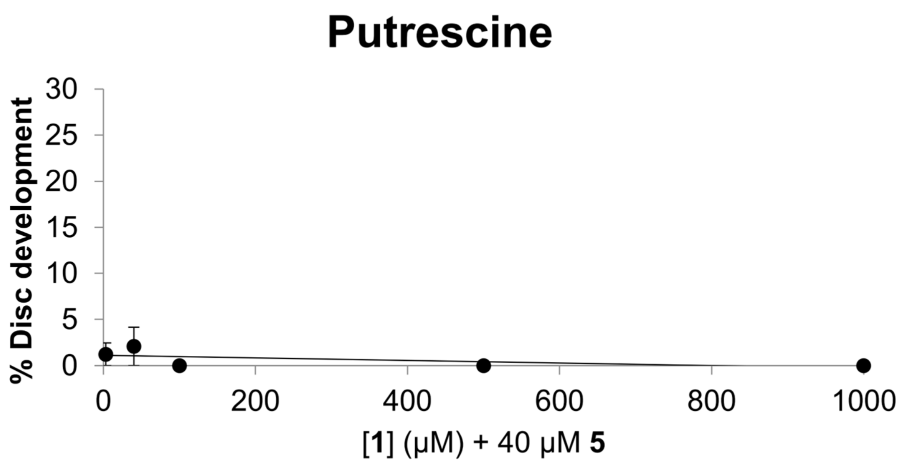

Figure S1. Putrescine fails to block the inhibitory effect of Ant44 (**5**) on imaginal disc development. Putrescine (**1**) was tested at different concentrations in the presence of 40 µM Ant44 (**5**) and the percentage of imaginal disc development was recorded for each concentration. Every data point was repeated at least in triplicate. Error bars reflect the standard error of the mean (SEM). An EC_{50} value was not calculated as putrescine was unable to block the inhibitory effect of Ant44 (**5**) on imaginal disc development.
